# Diversity of Iron Oxidizers in Groundwater-Fed Rapid Sand Filters: Evidence of Fe(II)-Dependent Growth by *Curvibacter* and *Undibacterium* spp.

**DOI:** 10.3389/fmicb.2018.02808

**Published:** 2018-12-03

**Authors:** Arda Gülay, Yağmur Çekiç, Sanin Musovic, Hans-Jørgen Albrechtsen, Barth F. Smets

**Affiliations:** ^1^Department of Environmental Engineering, Technical University of Denmark, Kongens Lyngby, Denmark; ^2^Department of Organismic and Evolutionary Biology, Harvard University, Cambridge, MA, United States; ^3^Department of Environmental Engineering, Istanbul Technical University, Istanbul, Turkey; ^4^Danish Technological Institute, Taastrup, Denmark

**Keywords:** iron oxidizing bacteria, novel, rapid sand filters, *Curvibacter*, *Undibacterium*, iron metabolism, *Ferriphaselus*, *Gallionella*

## Abstract

Although earlier circumstantial observations have suggested the presence of iron oxidizing bacteria (IOB) in groundwater-fed rapid sand filters (RSF), ferrous iron (Fe(II)) oxidation in this environment is often considered a chemical process due to the highly oxic and circumneutral pH conditions. The low water temperature (5–10°C), typical of groundwaters, on the other hand, may reduce the rates of chemical Fe(II) oxidation, which may allow IOB to grow and compete with chemical Fe(II) oxidation. Hence, we hypothesized that IOB are active and abundant in groundwater-fed RSFs. Here, we applied a combination of cultivation and molecular approaches to isolate, quantify, and confirm the growth of IOB from groundwater-fed RSFs, operated at different influent Fe(II) concentrations. Isolates related to *Undibacterium* and *Curvibacter* were identified as novel IOB lineages. *Gallionella* spp. were dominant in all waterworks, whereas *Ferriphaselus* and *Undibacterium* were dominant at pre-filters of waterworks receiving groundwaters with high (>2 mg/l) Fe(II) concentrations. The high density and diversity of IOB in groundwater-fed RSFs suggest that neutrophilic IOB may not be limited to oxic/anoxic interfaces.

## Introduction

Rapid sand filters (RSFs) receive continuous inputs from groundwater sources and this input may vary depending on the temporal and spatial dynamics of the aquifer (Gülay et al., 2016). Ferrous iron [Fe(II)] is a commonly occurring constituent in anaerobic groundwaters. Fe(II) concentrations vary significantly between the aquifers depending on the specific geological conditions (Søgaard and Madsen, 2013). Presence of Fe(II) in source waters requires a strict treatment to produce drinking water, as iron can cause bad taste, discoloration, turbidity and can support microbial growth in distribution systems (Sharma et al., 2005). Removal of Fe(II) often involves chemical oxidation by intensive aeration, and subsequent removal of iron oxyhydroxide precipitates by RSF (Twort et al., 2000).

Biological contributions to ferrous iron removal are not typically considered significant in RSFs. However, low water temperatures (<10°C), characteristic of groundwaters, retard chemical Fe(II) oxidation rates (Sung and Morgan, 1980), which may allow microbiological iron oxidation simultaneously with chemical oxidation (Gülay et al., 2013). Hence, these lower temperatures could provide a niche for iron oxidizers, in addition to the previously reported niches such as acidic (Heinzel et al., 2009) and anoxic (Straub et al., 2004) environments, as well as oxic/anoxic interfaces (Sobolev and Roden, 2001). It is challenging to measure the individual contributions of chemical and microbial processes to iron oxidation (Neubauer et al., 2002). Therefore, the presence and abundance of IOB has been widely used as an indicator for microbiological iron oxidation in environments such as groundwater/surface water interfaces (Yu et al., 2010), wetlands (Wang et al., 2009), hydrothermal vents (Emerson and Moyer, 2002), and acid mine waters (Heinzel et al., 2009). In these studies, IOB were retrieved by cultivation based techniques (for example, Emerson et al., 1999; Küsel et al., 2003; Krepski et al., 2012). While few indirect observations have suggested a microbial contribution to iron oxidation in RSFs (Mouchet, 1992; Søgaard et al., 2000; Katsoyiannis and Zouboulis, 2004), only three studies to date have reported the presence of putative IOB via cultivation (Gülay et al., 2013), 16S rRNA based microbiome sequencing (Gülay et al., 2016), or molecular enumeration of *Gallionella* spp. (de Vet et al., 2012). These studies have shown the presence and possible role of IOB in RSFs. Yet, an extensive picture of IOB diversity and abundance has remained elusive.

The Fe(II)/O_2_ gradient method (Emerson and Moyer, 1997) has been the typical technique to enrich and isolate circumneutral IOB. Using this method, many mixotrophic, hetetrophic and lithoautotrophic IOB belonging to the genera *Gallionella*, *Sideroxydans*, *Pseudomonas*, *Acidovorax*, *Rhodoferax*, and *Mariprofundus* have been isolated (reviewed in Emerson et al., 2010). Our previous study on RSFs revealed *Burkholderiales* dominating the Fe(II)/O_2_-enrichments (Gülay et al., 2013). We hypothesized that they may be involved in iron oxidation in RSFs.

In this study, we characterized the Fe(II) oxidizing guilds in pre- and after filter units at three different drinking water treatment plants (DWTPs) in Denmark. First, we confirmed the presence of IOB in RSFs based on evidence of cell-associated iron oxide sheaths. Using IOB enrichments, we then assessed total IOB abundance and subsequently described the phylogenetic composition of cultures found in the highest positive dilutions. We further used micro-manipulation to continue Fe(II)/O_2_ enrichments and examined Fe(II) dependent growth of putative IOB. We further designed FISH probes and monitored the growth of identified IOB in liquid incubations. Finally, the relative abundance of the newly identified and well-known IOB in the full-scale RSFs was assessed by inspecting the community bacterial 16S rRNA gene amplicon libraries (Illumina MiSeq and 454) against FISH-verified full 16S rRNA clones from IOB enrichments.

## Materials and Methods

### Sampling Sites and Groundwater Quality

Filter samples were obtained from three geographically distant DWTP in Denmark (Supplementary Figure S1): Islevbro DWTP (55°42′14″ N, 12°27′21″ E); Esbjerg DWTP (55°33′46″ N, 8°28′56″ E); Aike DWTP (55°28′20″ N, 8°46′11″ E). In Islevbro DWTP the groundwater is subjected to RSF with the following treatment chain: aeration, prefiltration in a rock filter bed (particle diameter 10–20 cm), and after-filtration in 12 sand bed filters with coarse grained sand (Supplementary Figure S2b; particle diameter 2–5 mm) overlaying a supporting gravel medium layer (Supplementary Figure S2a). Aike DWTP has an identical process flow as Islevbro DWTP, but both pre and after filters are pressurized RSFs (Supplementary Figure S3) packed with coarse grained sand (particle diameter 1–5 mm). Pre and after-filter units at Esbjerg DWTP are conventional RSFs (Supplementary Figure S4b) packed with coarse-grained sand (particle diameter 1–5 mm). Pre-filters of Esbjerg DWTP were designed to remove Fe(II) biologically. Influent anaerobic groundwater is first slightly oxygenated (1–2 mg/l) using cascade aeration and fed to the pre-filters (Supplementary Figure S4a). This provides an oxic/anoxic interphase and micro-aerophilic conditions favorable for IOB. Both biologically and chemically oxidized Fe(II) are then removed with backwashing. Effluent water from the pre-filter is then saturated with air in an aeration tank after pH adjustment with Ca(OH)_3_ or CO_2_. Chemistry of groundwater, influent water and effluent water of the investigated DWTPs are given in Supplementary Table S1.

Filter samples for IOB enrichments and DNA extraction were collected from pre- and after-filters. Samples (0–10 cm of top filter layer) were collected from three random positions of selected RSFs and subsequently combined in a sterile plastic bag. No samples could be taken from the pre-filters of Islevbro. Samples were directly transferred to laboratory on ice for further analysis.

### DNA Extraction

A 0.5 g combined drained sand filter sample and 0.5 ml suspensions of selected Fe(II)/O_2_ enrichments and liquid incubations were subjected to genomic DNA extraction using the MP FastDNA^TM^ SPIN Kit (MP Biomedicals, LLC., Solon, OH, United States) per manufacturer’s instructions at room temperature. The concentration and purity of extracted DNA was checked by NanoDrop 2000 spectrophotometer (NanoDrop Technologies, Wilmington, DE, United States).

### Most Probable Number (MPN) and qPCR Enumeration

Most probable number (MPN) was used to estimate the density of cultivable IOB in filter samples from pre- and after-filters. Ten-fold dilution series of samples were made in sterile modified Wolfe’s mineral medium (MWMM) (Emerson and Moyer, 1997) and inoculated in Fe(II)/O_2_ gradient tubes (see below). All MPN media were run in triplicate, incubated at 10°C for 20 days. Microbial growth in gradient tubes of serial dilutions were confirmed with microscopic examination with SYTO9 staining and the IOB density in original samples was estimated according to Jarvis et al. (2010).

Quantification of *Gallionella* types was done by quantitative polymerase chain reaction (qPCR) on total DNA extracted from the filter samples collected from three DWTP using a *Gallionella* specific 16S rRNA gene targeted primer set (Heinzel et al., 2009). qPCR analyses were conducted in a Chromo4 thermocycler operated by the Opticon Monitor 3 370 software (BioRad). Each qPCR reaction contained 12.5 μl 2× iQ SYBR Green Supermix (BioRad Laboratories), 10 pmol primer, DNA template (10 ng) and DNA/RNAfree water (Mol. Bio.) to 25 μl. The thermal cycling conditions consisted of an initial 15 min denaturation at 95°C followed by 40 cycles of: 15 s at 95°C; primer annealing at 20 s at 55°C and 25 s extension at 72°C. Amplification specificity was examined by melting curve analysis (gradient 0.2°C/s, range 70–95°C) (Ririe et al., 1997). MPN and qPCR results were normalized to the mass of drained sand material and the results (cell/gr Wet sand and gene/gr Wet sand) were shown as cell/gr and gene/gr throughout the manuscript, respectively.

### Gradient Cultivation and Advanced (Further) Enrichments

Filter sand samples (approximately 3 g wet weight) were suspended in 5 ml Wolfe’s mineral medium (MWMM) at pH 7.10 and cells were dislodged by shaking the mixture at 150 rpm for 30 min. Supernatant was used as inoculum for enrichment experiments and MPN enumeration. The opposing Fe(II)/O_2_ gradient technique (Kucera and Wolfe, 1957) was used as cultivation method and implemented according to Emerson and Floyd (2005).

The two-layered growth medium (volume 6.75 ml, at pH 7.1) in a 11 ml glass tube was established by including a 0.15% (wt/vol) agarose (low melt agarose; Sigma-Aldrich), 1X MWMM medium, Wolfe’s vitamin and trace element solution ^1^ (MDVS, MDTMS) and 10 mM sodium bicarbonate at the upper layer, and 1% agarose stabilized plug of freshly synthesized FeS (Emerson and Floyd, 2005) together with 1X MWMM medium at the bottom layer (volume 1.25 ml). The inoculation was performed by slowly expelling the 10 μl inoculum from bottom to the top in the upper medium of a gradient tube. Abiotic controls were prepared without microbial inoculation. The gradient tubes were kept at 10°C for 24 h prior to the inoculation to allow formation of Fe(II) and O_2_ gradients. All enrichments were incubated in the dark at 10°C.

Growth was determined using microscopic examination with SYTO9 staining and visual inspection by detecting iron oxide bands located at different locations (typically much higher) than those in control tubes. For the first phase of the phylogenetic analysis, bacterial cells from highest positive dilution of MPN series were collected and subject to DNA extraction and DGGE (Supplementary Figures S5, S6 and Supplementary Table S3). In addition, six of the highest positive dilutions were transferred to liquid incubations, to attempt to validate Fe(II) dependent metabolism. With this experiment, we were not able to validate the iron metabolism, because significant diversity loss was detected between tube transfers (Wang et al., 2009) and similar DNA and cell counts were obtained from both negative and positive controls (Supplementary Figure S8). This led us to develop an advanced enrichment using a micro-manipulation technique.

Distinct locations, above clear IOB bands were detected. The original Fe(II)/O_2_ gradient tubes were micro-subsampled (2 μl) using a micro pipette tip. Obtained microsamples were used as inoculum for the new Fe(II)/O_2_ gradient tubes (Figure 2 (3)). A subsequent retransfer step was applied to these successive advanced enrichments (12 units; Figure 2 (4)) before they transferred to liquid incubations.

### Liquid Batch Incubations

To select putative and novel IOB, a liquid incubation technique was first applied to (i) six highest positive dilutions of the original MPN series (Figure 2 (3)), then (ii) 12 advanced (further) enrichments (Figure 2 (5)), and finally (iii) 10 cell extracts of RSF materials (Figure 2 (6)) as described by Emerson and Moyer (2002). For each inoculum, two parallel batch incubations were prepared with the identical media (without agarose) in sealed 25 ml serum bottles at pH 7.1 (Figure 2 (5)): (1) The liquid incubations for all batches were supplied with 0.04 ml 100 mM FeCl_2_ and 0.8 ml sterile air at 24 h intervals, and (2) negative (abiotic) controls were solely supplied with 0.8 ml sterile air at the same time intervals. To minimize heterotrophic microbial growth, no external organic carbon source was added. The serum bottles were incubated for 15 days at 10°C in the dark without agitation. Liquid incubations were daily sampled to measure cell growth. At the end of 15 days incubation, total biomass was extracted from all incubations using a sterile 0.2 μm pore size (Millipore, Billerica, MA, United States) membrane filter prior to the DNA extraction. In the last (iii) liquid incubation experiments (from cell extracts of RSF materials) FISH samples were collected at 3 days intervals during 15 days and were immediately fixed in 4% paraformaldehyde after biofilm sampling. A total of 10 replicate incubations and 2 controls were run and examined with 4 newly-designed FISH probes (Supplementary Table S4; see *FISH experiments*). The total number of cells in inocula and daily samples of liquid incubations were estimated by direct microscopic counting (TDC) of (Syto9, Molecular Probes, Inc., Eugene, OR) stained cells using Confocal Scanner Laser Microscopy (CLSM, Leica TCS SP5, Leica Microsystems, Wetzlar, Germany), to estimate the cell densities within the incubations and allow a better quantification of bacterial cells enmeshed in a matrix of iron oxides (Gülay et al., 2014). The acquired CSLM images were subject to image analysis software (Image Pro Plus 4.1).

### Denaturing Gradient Gel Electrophoresis (DGGE)

Extracted DNA from the cultures of highest positive dilutions of MPN series and liquid incubations of 12 advanced Fe(II)/O_2_ gradient enrichments were amplified using the bacterial specific primer set 341FGC and 518R (Muyzer et al., 1993) with PCR conditions described in Gülay et al. (2013). DGGE analysis of the amplicons was performed as described by Gülay et al. (2013) by using the Dcode system (BioRad, Hercules, CA, United States) with 8% (v/v) polyacrylamide gels and a denaturant gradient of 35–53%. DGGE profiles were visualized and images recorded by the GelDoc (BioRad). Individual DGGE bands were cut out from the gel, incubated overnight at 4°C in 25 ml H_2_O, reamplified (without GCclamp), purified using Qiagen PCR purification kit (Qiagen, Valencia, CA, United States) and subject to sequencing. Band comparisons between DGGE profiles were performed using BioNumerics v4.0 software (Applied Maths). Bands were defined for each sample using the band search algorithm with position tolerance of 1%. Highest positive dilution samples having only a single band (15 samples) were cloned and sequenced using the 27F and 1492R primer set to obtain nearly full length 16S rRNA gene of the DGGE detected phylotypes (Supplementary Table S3).

### Cloning and Amplified Ribosomal DNA Restriction Analysis (ARDRA)

Bacterial 16S rRNA genes from liquid incubations of 12 advanced Fe(II)/O_2_ gradient tube enrichments were PCR amplified, cloned as described by Sudek et al. (2009). 45 clones were obtained for each incubation and clones were further screened by ARDRA technique using restriction enzymes HhaI, RsaI, and HaeIII (Invitrogen, Carlsbad, CA, United States). Unique clones were subjected to sequencing after plasmid isolation (Qiagen plasmid mini kit). M13F/M13R plasmid targeted primers were used for two-sided sequencing of plasmid DNA.

### Probe Design and FISH Experiments

FISH probes targeting 16S rRNA gene were designed and evaluated using the probe design and probe match algorithms of ARB (Ludwig et al., 2004). The 16S rRNA database of SSU-Ref_NR99_132 (Quast et al., 2013) was downloaded and updated by importing all high quality and almost full-length 16S rRNA cloning sequences obtained in this study. The *Ferriphaselus*, *Curvibacter*, and *Undibacterium* genera were defined based on SILVA classification and sequences forming a stable monophyletic group. *Curvibacter fontana*, *Ferriphaselus amnicola*, and uncultured representatives of *Undibacterium parvum* were selected and used to design *Ferriphaselus*, *Curvibacter*, *Undibacterium* specific probes (Figure 4; colored strains). For the *Rhodoferax* probe, published *Rhodoferax* sequences together with one clone sequence were targeted. Hybridization thermodynamics of the designed FISH probes were evaluated with *in silico* simulations using mathFISH (Yilmaz et al., 2011; Supplementary Figure S9). Optimal hybridization conditions were experimentally determined as described previously (Daims et al., 1999). The conditions yielding bright fluorescence signals for the target organisms at the highest formamide concentration in the hybridization buffer were experimentally assessed and used for the designed FISH probes (Supplementary Figure S10) FISH experiments were executed with multiple probes having different fluorochromes to identify unambiguously the target organisms according to the multiple probe concept (Lücker et al., 2014). *E. coli* MG1655 was used as a control to validate the probe specificity. All FISH probes used in this study are listed in Supplementary Table S4.

Samples from liquid incubations inoculated with enrichments of cell extracts of RSF materials (Figure 2 (6)) were collected with 3 day intervals during 15 days of operation with a syringe. Specimens containing visible microbial mass were fixed in 4% paraformaldehyde. Fixed samples were dehydrated and sequentially probed with Fluorescein (FLUO), Cy3- and Cy5- tagged 16S rRNA probes (Sigma-Aldrich, St. Louis, MO, United States; Supplementary Table S4), following procedures described elsewhere (Pellicer-Nàcher et al., 2014). Growth was further studied by SYTO 90 (Invitrogen, Carlsbad, CA, United States) following manufacturer’s specifications. Hybridized and stained specimens were inspected with a confocal laser-scanning microscope (CLSM, Leica TCS SP5, Leica Microsystems, Wetzlar, Germany) equipped with an Ar laser (488 nm), and two HeNe lasers (543 and 633 nm). At least five micrographs retrieved from specimens hybridized with probe combinations *Undibacterium* & *Rhodoferax* and *Curvibacter* & *Ferriphaselus* (Supplementary Table S4) were taken using the image analysis software Daime (Daims et al., 2006).

### Amplicon Sequencing

Ten ng of extracted DNA from combined filter samples taken from DWTP were PCR amplified using Phusion (Pfu) DNA polymerase (Finnzymes, Finland) and 16S rRNA gene targeted modified universal primers 341F and 806R (Yu et al., 2005). PCR was performed as described elsewhere (Gülay et al., 2016) and sequencing was performed at the DTU Multi Assay Core Center (Kongens Lyngby, Denmark).

### Bioinformatics and Phylogenetic Analysis

High quality DGGE sequences were selected from chromatograms using BioEdit 7.0.9.0 software, manually trimmed and transferred to web-based SINA v1.2.11 (Pruesse et al., 2007) for alignment and selection of top 10 closest relatives for each DGGE sequence. Aligned sequences were then imported into the ARB software environment (Ludwig et al., 2004) and the alignments were further edited manually. Raw sequences from cloning and sequencing were processed as described above. DGGE sequences that belong to the putative IOB (white circles; Figure 5) were added to the clone library of advanced enrichments.

All raw 16S rRNA amplicons from 454 pyrosequencing were denoised, processed and classified using the QIIME^2^ software package (Caporaso et al., 2010). Raw 16S rDNA amplicons from MiSeq were denoised with Deblur (Daims et al., 2006), processed and classified with QIIME and Mothur (Schloss et al., 2009; Caporaso et al., 2010). Chimera checking was performed with the software UChime (Edgar et al., 2011). High quality sequences were clustered at 100% evolutionary similarity. For taxonomic classification and relative abundance analysis, the Silva128 database was modified by adding nearly full length 16S rRNA sequences of IOB, that were targeted by FISH probes of *Curvibacter*, *Undibacterium* and *Ferriphaselus*, within the clone library (Figure 4). Relative abundances of novel IOB were determined by blasting FISH-verified nearly full 16S amplicons against Miseq and 454 libraries and relative abundances of known IOB were determined by blasting published IOB isolates against Miseq and 454 libraries. Results shown are average of Miseq and 454 libraries.

### Nucleic Acid Sequences

Raw SFF files from Miseq Illumina run were deposited into the Sequence Read Archive at GenBank under the study accession number SRR7969367. 16S rRNA clone sequences generated in this study were deposited in the GenBank under accession numbers MK000735 to MK000880. 454 pyrosequencing sequences were deposited into the Genbank under sample numbers SAMN10176812–SAMN10176818.

### Environmental Scanning Electron Microscopy (ESEM)

A FEI Quanta 200 F ESEM FEG microscope was used for ESEM investigations. Pore water, obtained by slow centrifugation (1,800 *g*, 10 min) of the filter materials from an after filter Islevbro DWTP (Gobet et al., 2012), was mounted on an aluminum trap and imaged at 10 mbar partial water pressure (Gülay et al., 2014).

## Results

### Water Characteristics and Descriptions of the Investigated DWTP

Three geographically separated conventional full-scale DWTPs (Supplementary Figure S1), Islevbro (Supplementary Figure S2), Esbjerg (Supplementary Figure S3), and Aike (Supplementary Figure S4), were investigated in this study. In general, groundwaters from aquifers in Denmark are similar in terms of temperature (8–10°C), pH (6.9–7.2), and dissolved oxygen content (0–0.53 mg/l) (Klaus, 2003). In Esbjerg DWTP, influent Fe(II) concentrations are 10-fold higher than at other DWTPs (12 mg/l; Supplementary Table S1). All other measured parameters were at similar levels between DWTP.

Treatment train in all DWTPs consist of an aeration unit and two filtration units: pre- and after-filter. Pre-filters of Esbjerg DWTP were designed for biological Fe(II) oxidation by controlling the pH between 6.5 and 7 and holding the dissolved oxygen concentrations low (1–2 mg/l O_2_; Supplementary Figure S4). Orange-brown iron blankets were observed at all pre- and after-filter beds and brown iron oxides were observable down to a depth of 10 cm below the filter surface.

### Evidence of IOB Occurrence and Quantification of Total IOB and *Gallionella* spp. Abundance

**FIGURE 1:**
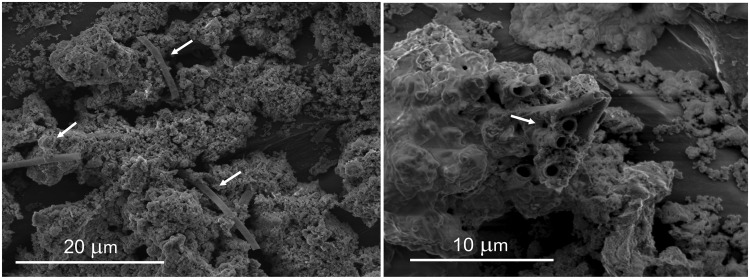
Environmental scanning electron microscope (E-SEM) images of materials extracted from pore water from after-filter sand grains at the Islevbro DWTP. Biogenic remains (arrows) are visible between the mineral Fe(III) oxyhydroxide precipitates.

**FIGURE 2:**
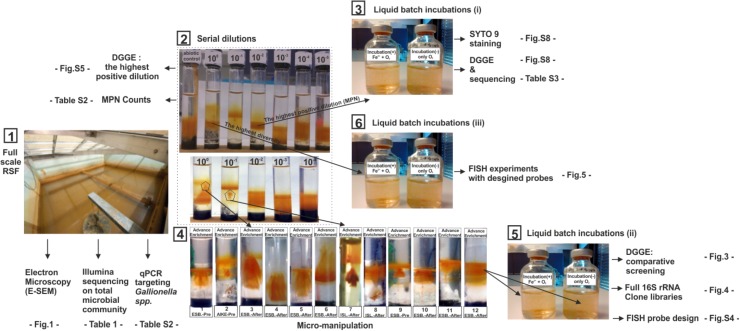
Overview of experimental sequence and methods used in this study. (1) Experiments used cell extracted from filter materials recovered from full-scale RSFs (2) A representative set of Fe^2+^/O_2_ opposing gradient serial dilution tubes for IOB MPN enumeration and enrichments. First tube on the left is a control tube without cell inoculation; tubes labeled from 10^0^ to 10^-5^ are inoculated with 10 μl of 10-fold serially diluted cell suspensions. (3) Verification of Fe^2+^ dependent growth in liquid cultures by comparing results of DGGE and Syto-9 staining of incubations with and without Fe^2+^ amendment (4). Microsampling at distinct locations of the IOB enrichment band in the original Fe^2+/^O_2_ gradient tube (5) Verification of Fe^2+^ dependent growth in liquid cultures by comparison of DGGE, clone libraries and (6) FISH images of incubations with and without Fe^2+^ amendment. FISH probes were designed from the clone library constructed at (5).

Environmental scanning electron microscopy (ESEM) micrographs on extracted materials from pore waters of filter grains (Figure 1) of Islevbro DWTP revealed the presence of sheaths, which are characteristic of cultivated IOB such as *Leptothrix discophora* (Emerson and Ghiorse, 1992) or *Mariprofundus ferrooxydans* (Emerson et al., 2007). A first effort at determining total IOB density was performed by MPN based quantification with Fe(II)/O_2_ enrichments. Culture based estimates of IOB abundance (Figure 2), in the three DWTP ranged from 1.80 × 10^4^ to 6.00 × 10^7^ cells/gr in pre-filters and from 1.10 × 10^4^ to 2.30 × 10^6^ cells/gr in after-filters (Supplementary Table S2). The highest IOB cell numbers were detected in the pre- and after-filters at Esbjerg DWTP. The lowest IOB cell numbers were measured in the after-filters at Islevbro DWTP, where the lowest influent ferrous iron concentration was observed. The IOB densities at after-filters of Aike and Islevbro DWTPs were higher than at the pre-filters, while in the pre-filters of Esbjerg DWTP, where biological iron oxidation was enhanced by low dissolved oxygen concentrations, cell numbers were an order of magnitude higher than in the after-filter.

16S rRNA gene-based qPCR abundance estimates were consistently one or two orders of magnitude higher than the cultured based MPN estimates. Densities of *Gallionella* spp. in pre-filters, based on qPCR, ranged from 3.07 × 10^7^ to 9.92 × 10^8^ gene/gr while in after filters its abundances ranged from 2.83 × 10^6^ to 6.76 × 10^8^ gene/gr. In line with the MPN, qPCR results displayed highest abundance of *Gallionella* spp. at pre- and after-filters at Esbjerg DWTP.

### Identification of Putative Iron Oxidizers From Fe(II)/O_2_ Enrichments

#### Fe(II)/O_2_-Gradient and Liquid Enrichments

Highest positive dilutions of MPN series were fingerprinted using DGGE (Muyzer et al., 1993). The 16S rRNA DGGE sequences obtained from the 56 highest dilutions (Supplementary Figure S6) belonged to the clades *Betaproteobacteria*, *Gammaproteobacteria*, and *Bacteriodetes*. Sequences within *Gammaproteobacteria* and *Bacteriodetes* phyla affiliated to *Pseudomonas* and *Flavobacterium*, respectively. These taxa were mostly detected in the filters of Aike and Islevbro DWTP. Sequences in the *Betaproteobacteria* phylum branched within the *Comamonadaceae* and *Oxalobacteraceae*. These families were detected in all units of investigated DWTPs.

In 15 out of the 56 enrichments, a singular dominant DGGE band was detected (Supplementary Figure S5; marked as “+”), suggesting dominance of a single taxon. These 15 samples were further characterized by cloning and sequencing targeting 16S rRNA gene (Supplementary Table S3). Clone sequences from enrichments with singular dominant DGGE bands were distributed within the clades of *Curvibacter*, *Janthinobacterium*, *Rhodoferax*, *Massilia*, *Hydrogenophaga* (Supplementary Table S3). In addition, two strains of the genus *Undibacterium* (from Esbjerg) and *Herminiimonas* (from Aike and Islevbro) were detected.

### Further Evidence on Fe(II) Based Growth of Putative Iron Oxidizers

#### Advance Fe(II)/O_2_-Gradient Enrichments

The long incubation period in the Fe(II)/O_2_-gradient tubes (>12 days) allows non-Fe(II) dependent organisms to grow together with, and supported by IOB (Yu et al., 2010). While the final Fe(II)/O_2_-gradient tubes were obtained from the last positive serial dilutions and after several re-transfers, which substantially reduces the non-Fe(II) dependent diversity (Wang et al., 2009; Supplementary Figure S5), to minimize the growth of non-IOB in subsequent enrichments, a further IOB selection was applied by micro-sampling (2 μl) IOB bands where clear biogenic iron oxides could be identified (Figure 2 (4)). A total of 12 further enrichments were successfully obtained showing different Fe(III)-oxide structures (Figure 2 (4)).

#### Liquid Incubations and Taxonomic Identification

**FIGURE 3:**
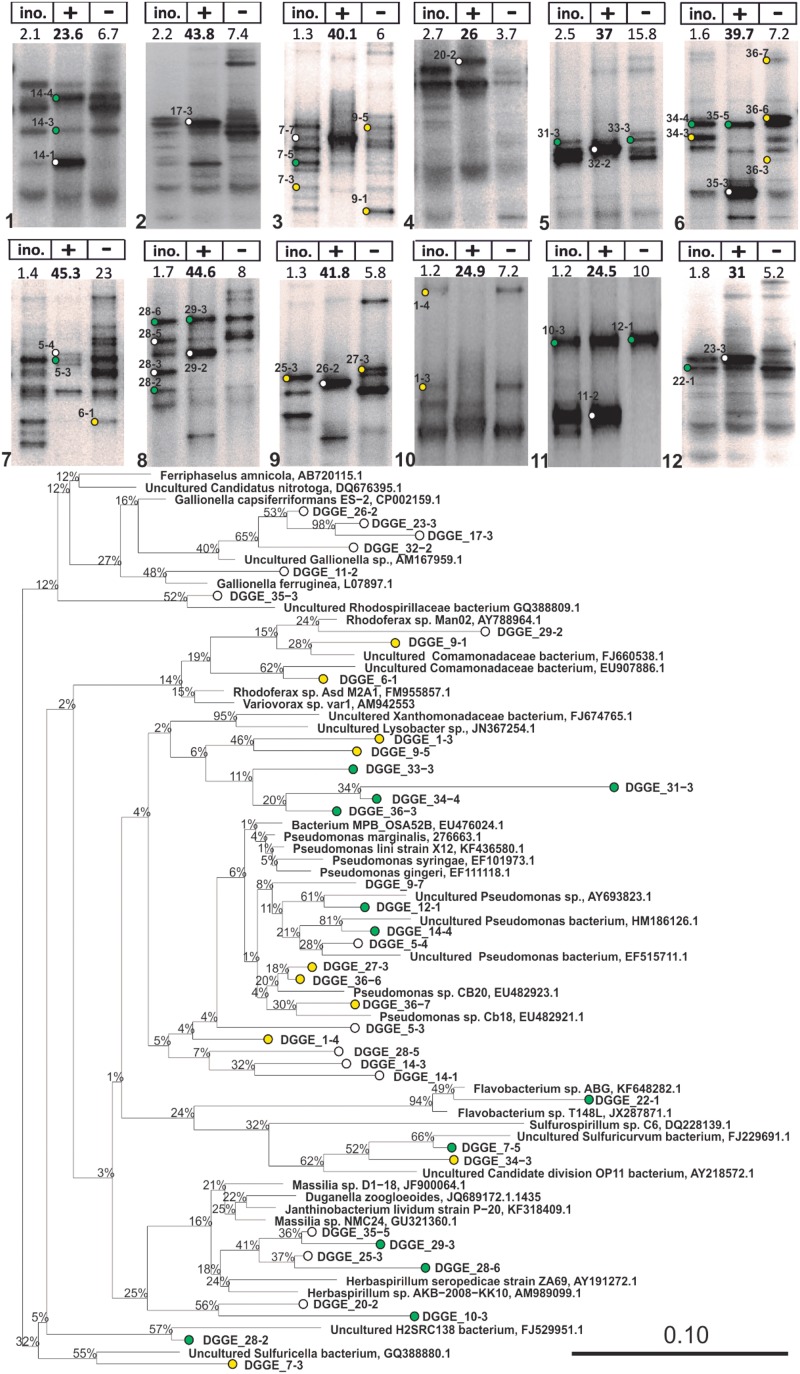
16S rRNA DGGE profiles of advanced IOB enrichments taken from liquid cultures with (+) and without (–) Fe^2+^ addition (Step 5 in Figure 2) and phylogenetic tree of selected 16S rRNA gene sequences obtained from isolated DGGE bands. The tree was constructed using neighbor-joining method in ARB after 1000 bootstrap iterations. Closely related sequences, with respective GenBank accession numbers, are shown as reference. White, green, and yellow dots are sequences from bands that were solely detected in incubations with Fe^2+^, detected in both incubations with and without Fe^2+^, and solely detected in incubations without Fe^2+^, respectively. Scale bar indicates 10% sequence difference.

In each of the 12 advanced enrichments, IOB growth resulted in unique brownish Fe(III)-oxide structures (Figure 2 (4)). In addition, microbial growth was confirmed in these gradient tubes by direct cell count with CSLM and DNA measurements applied before and after the enrichment period. Aliquots from the advanced enrichments were then inoculated into liquid media (termed liquid incubations) with and without Fe(II). In incubations with Fe(II) amendment, substantial and consistent growth was observed resulting in 11- to 32-fold increase over the initial DNA concentrations, and 2- to 7-fold higher DNA values than in the control incubations (Figure 3).

Advanced Fe/O_2_ enrichments and liquid incubations were first assessed by 16S DNA targeted DGGE (Supplementary Figure S7), showing a clear difference in DGGE profiles of Fe(II) fed- and unfed-incubations (Figure 3) and the inoculum. Undetectable levels of PCR products (DGGE lanes without bands in Supplementary Figure S7) were obtained from DNA extracted from the liquid incubations without Fe(II) amendment, after 35 PCR cycles (Gülay et al., 2013). Only by increasing template concentration and number of PCR cycles could DGGE profiles be obtained; detected taxa were recognized to be non-IOB guilds.

To identify putative Fe(II)-dependent phylotypes, DGGE bands were grouped into three categories (Figure 3): bands that (i) solely occurred in incubations with Fe(II), (ii) occurred in both incubations, and (ii) solely occurred in incubations without Fe(II). Phylotypes that solely occurred in Fe(II) fed incubations were related to *Gallionella capsiferriformans*, *Gallionella ferruginea*, *Rhodoferax* spp., and *Herbaspirillum* spp., as well as the *Rhodospirillaceae* family (Figure 3). Phylotypes only present in incubations without Fe(II) were related to *Xanthomonadaceae*, and *Pseudomonas*. These strains are considered non-IOB in this study.

**FIGURE 4:**
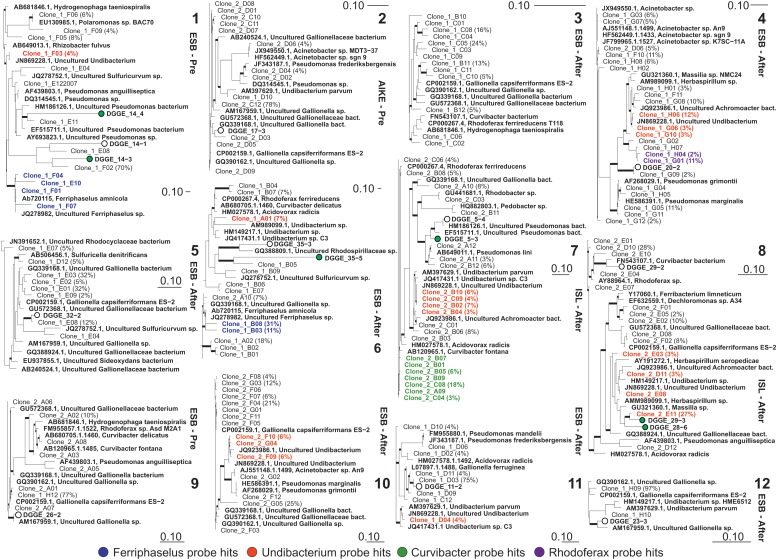
Phylogenetic trees of bacterial 16S rRNA gene sequences retrieved from incubations with Fe(II) addition after 15 days incubation (Numbers as in Figure 3; see Figure 2 step 5). The tree was constructed using neighbor-joining method in ARB from full length sequences obtained via cloning and partial sequences (170 bp) obtained from excised DGGE bands. Percentages in parentheses correspond to the relative abundance of clones with identical ARDRA profiles; remaining clones represented as single OTU in corresponding ARDRA profiles. Thick branches on phylogenetic trees indicate high parsimony bootstrap support (≥70%) based on 1000 iterations. Scale bars indicate 10% sequence difference.

In addition to the DGGE library, nearly full length 16S rRNA clone libraries of communities from the 12 Fe(II)-amended liquid incubations (Figures 2 (4,5)) were constructed (Figure 4). The majority of the *Betaproteobacterial* clones affiliated with the genera *Gallionella*, *Acidovorax*, *Ferriphaselus*, *Undibacterium*, and *Curvibacter* (Figure 4). The remaining clones belonged to the *Rhodoferax*, *Sulfuricurvum*, *Pseudomonas*, and *Acinetobacter*. *Gallionella* spp., as well-known IOB, were primarily detected in the pre-filters at the Esbjerg DWTP and in the pre-filters at Aike DWTP; they were not dominant (Figure 4) in after-filter of Islevbro DWTP.

*Gallionella* spp. dominated the clone libraries excluding libraries #4 and #7 (after filters of Esbjerg and Islevbro). The clone library of liquid batch incubation #4 showed that *Undibacterium* dominated the microbiome at 31% of the total community density without detectable presence of *Gallionella* or *Ferriphaselus*. Second, *Curvibacter* and *Undibacterium* strains dominated the clone library of liquid batch incubation #7 at 20 and 30% of the total community, respectively while *Gallionella* types were detected at only 8%.

### Validation of Novel Iron Oxidizers

**FIGURE 5:**
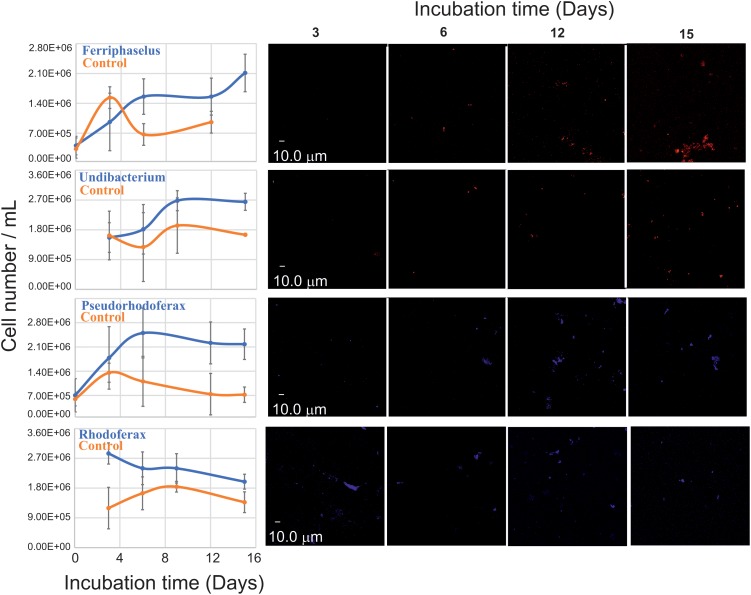
Quantitative FISH analysis against putative IOB (left) during batch liquid incubations (Figure 2, step 6) with (blue) and without (red) Fe^2+^ amendment using probes developed in this study (Supplementary Table S4). For each data point, the mean cell number of 10 biological replicates was measured from 10 FISH micrographs per replicate. Error bars indicate the standard deviation. Representative FISH micrographs (right) of applied probes at different time points of batch incubations.

Since *Undibacterium* and *Curvibacter sequences* were consistently detected together with sequences of known IOB, *Gallionella* and *Ferriphaselus*, we designed 16S rRNA-targeted probes for the FISH based specific detection of *Undibacterium* spp., and *Curvibacter* spp. according to the multiple probe approach (Ludwig et al., 1998; Figure 4). We applied FISH on time-series samples from liquid batch Fe(II)/O_2_ incubations to examine cell growth of *Undibacterium* and *Curvibacter* on Fe(II), as the sole energy source (Figure 5).

Two additional FISH probes targeting *Rhodoferax* and *Ferriphaselus* phylotypes, were designed: *Ferriphaselus* is a known Fe(II) oxidizer (Kato et al., 2014) and serves as positive control (i.e., growth is expected) yet no Fe(II) oxidation metabolism has been reported for *Rhodoferax*. *Rhodoferax* probe was designed to cover published *Rhodoferax* strains. Hence, no growth was expected from *Rhodoferax* probe targeted phylotypes and serves as negative control.

FISH confirmed the growth of *Ferriphaselus* strains in Fe(II)/O_2_ incubations. Similar growth patterns were detected for types targeted with *Curvibacter* and *Undibacterium* probes and these phylotypes yielded higher cell numbers than *Ferriphaselus* strains at the end of the incubation period. Hence, Fe(II) dependent growth was confirmed for strains in the enrichment belong to *Ferriphaselus*, *Curvibacter*, and *Undibacterium* In contrast, FISH signals targeting *Rhodoferax* phylotypes consistently decreased over the incubation period; hence Fe(II) oxidation by *Rhodoferax* was not confirmed.

### Relative Abundance of Known and Novel IOB at Full-Scale Microbial Communities

To evaluate the relative abundances of known and novel IOB identified in this study in their source environment, 16S rRNA amplicon libraries of the source communities were analyzed for the presence of identified IOB in this study. The community libraries of total communities was blasted (Basic Local Alignment Search Tool) against nearly full-length 16S rRNA genes of known and novel (FISH verified) IOB that were obtained. Relative abundances of these strains followed a similar pattern as MPN counts, in that highest relative abundances were found in RSFs that receives highest Fe(II) concentrations and IOB densities decreased with decreasing influent Fe(II) concentration within and across DWTP (Table 1). *Undibacterium* strains were detected at highest densities in Aike DWTP (between 6 and 1.3% of the total community) while *Curvibacter* strains were below 1% at all DWTPs. The major known IOB lineage in the pre- and after-filters at all DWTPs belonged to the *Gallionella* spp. from the family Gallionellaceae. Relative abundance of *Gallionella* spp. decreased from pre- to after-filters. The *Acidovorax* lineage was the second most dominant known IOB at Islevbro DWTP (Table 1). Although *Leptothrix* sequences, a known freshwater iron oxidizer (Szewzyk et al., 2011) were not detected in enrichment clones and sequencing results, E-SEM suggested its presence in after filters of Islevbro DWTP.

**Table 1:** Relative abundance of known and novel IOB in investigated waterworks.

ID	Genus	Relative abundance				
		Aike		Esbjerg		Islevbro
		Pre	After	Pre	After	After
Novel IOB	*Undibacterium^1^*	**6% ± E00**	**1.33% ± E-01**	0.13% ± E-02	0.07% ± E-02	0.21% ± E-01
	*Curvibacter^1^*	0.15% ± E-01	0.17% ± E-01	0.4% ± E-01	0.08% ± E-01	0.10% ± E-01
Known IOB	*Ferriphaselus^2^*	**1.4% ± E-05**	0.05% ± E-04	0	0	0.01% ± E-05
	*Gallionella^2^*	**49.2% ± E+01**	**21.3% ± E+01**	**59.2% ± E+01**	**39.5% ± E+01**	**10.5% ± E+01**
	*Acidovorax^3^*	0.1% ± E-02	0.5% ± E-01	**1% ± E-01**	0.64% ± E-01	**1% ± E00**
Total IOB		56.9%	23.4%	60.7%	40.3%	11.8%


*Bold numbers indicate relative abundance values over >1%.*

*^1^Relative abundances were obtained by blasting FISH verified clones against Miseq and 454 libraries.*

*^2^Relative abundances were obtained by blasting FISH verified clones with well-known IOB isolates from Silva128 against Miseq and 454 libraries.*

*^3^Relative abundances were obtained by blasting well-known IOB isolates from Silva128 against Miseq and 454 libraries.*

## Discussion

### Diversity of Iron Oxidizers in RSFs

Previously, *Gallionella* and *Leptothrix* lineages were assumed to constitute the diversity of IOB in RSFs and quantifications of IOB in RSFs have been made using specific 16S rRNA gene primers (e.g., de Vet et al., 2012). Identifying IOB in environmental samples based on molecular approaches remains challenging (Yu et al., 2010) as previous studies have revealed large diversities based on Fe(II)/O_2_ enrichments (Emerson et al., 1999; Küsel et al., 2003; Krepski et al., 2012; Gülay et al., 2013). In this study, 150 nearly full-length 16S rRNA gene sequences were retrieved from 12 advanced enrichments that included modified Fe(II)/O_2_ gradient cultivation followed by selective liquid incubation. Multiple lines of evidence were provided to show Fe(II)-dependent growth of phylotypes affiliated to *Ferriphaselus*, *Curvibacter*, and *Undibacterium*. Two novel iron oxidizing taxa were identified within the *Curvibacter* and *Undibacterium* genera.

Several phylotypes of the *Gallionellaceae* were found in the enrichments with *Gallionella capsiferriformans* ES2, *Gallionella* strain R1 and *G. ferruginea* as closest cultured relatives. *G. ferruginea* is able to switch between autotrophic and hetetrophic life style (Hallbeck and Pedersen, 1991), which may provide a selective advantage in RSFs. Yet, most *Gallionella* phylotypes had uncultured representatives as closest relatives, as observed for other RSFs studies (de Vet et al., 2012). *Pseudomonadales* were not responsible for iron oxidation in investigated RSFs; closest relatives of the *Pseudomonas* phylotypes were uncultured strains retrieved from groundwaters.

Our results indicate that members of the *Undibacterium* and *Curvibacter* displayed iron oxidation dependent growth. Batch enrichment show that they were not (i) cross-feeding on decay or metabolic products of other known IOB such as *Gallionella* and *Ferriphaselus*, were not (ii) feeding on residuals of inoculum cells, nor were (iii) heterotrophs that fed on the agarose present in the gradient-tube media. *Undibacterium* or *Curvibacter* plus *Undibacterium* affiliated phylotypes dominated the clone libraries in some enrichments where no other typical IOB (*Gallionella or Ferriphaselus*) were detected. Control liquid incubations without Fe(II) addition did not support *Undibacterium* nor *Curvibacter* growth.

Further evidence was provided by FISH-based direct observation of cell-number increase under selective condition with Fe(II) was the sole energy source: growth and density curves of *Curvibacter* and *Undibacterium* were very similar to *Ferriphaselus*, a well-known IOB. At the end of the incubation period, *Curvibacter* and *Undibacterium* cell numbers were higher than *Ferriphaselus*, indicating metabolic independence from *Ferriphaselus.*

*Curvibacter fontana* sp., the closest relative of the identified *Curvibacter* phylotypes, was isolated from well water on heterotrophic growth medium (Ding and Yokota, 2010). Although heterotrophic media have been used to culture *Curvibacter* strains (Ding and Yokota, 2004, 2010), this clade is frequently detected in environments dominated by chemolithoautotrophy such as hydrothermal vents (Fortunato and Huber, 2016; Maltman et al., 2016), Fe(II) rich wetlands (Schmidt et al., 2014) and hot springs (Fortney et al., 2018), as well as groundwaters. So far *Curvibacter* isolates have been examined for degradation of simple organics (Ding and Yokota, 2004, 2010), but their chemolithoautotrophic has not yet been tested.

Little is known about the *Undibacterium* clade. *Undibacterium* strains have been isolated from various environments [waterfall (Du et al., 2015), shrimp culture pond (Sheu et al., 2014), soil and freshwater (Kim et al., 2014)], but the ability for chemolithotrophy has not been tested.

The possibility for autotrophic pathways in *Curvibacter* and *Undibacterium* strains was examined by checking for the presence of the ribulose-1,5-bisphosphate carboxylase/oxygenase (RubisCO) genes in published *Curvibacter* and *Oxalobacteraceae* (to which *Undibacterium* belongs) genomes and known IOB. RubisCO is the key enzyme of autotrophic CO_2_ fixation using the Calvin-Benson-Bassham cycle and has been widely used to explore the diversity of autotrophic organisms (Hügler and Sievert, 2011; Swan et al., 2011). We identified one *Curvibacter* genome, several *Oxalobacteraceae* genomes, as well as several *Gallionella* and *Mariprofundus* genomes that contain the RubisCo genes (Supplementary Figure S11). Hence, genes for autotrophy are present in *Curvibacter* and *Oxalobacteraceae*.

### Distribution of Iron Oxidizers in RSFs

At circumneutral pH, IOB are often reported to colonize oxic/anoxic interfaces of sediments in groundwaters, lakes and rivers (Hedrich et al., 2011). In this study, we report high densities and diversity of IOB in RSFs, which are highly oxic (>8 mg/l DO) and have circumneutral pH. Considering that the microbial colonization is mainly within the outer periphery (60.6 ± 35.6 μm) of the mineral coating of RSF materials (Gülay et al., 2014), it is unlikely that internal pores of RSF material have microaerophilic conditions that support IOB growth. Hence, IOB success may be related to the low water temperatures (<10°C) that would result in low chemical iron oxidation rates (Sung and Morgan, 1980; Gülay et al., 2013). Sung and Morgan (1980) observed an almost 100-fold increase in oxidation half-time of Fe(II) with a temperature decrease from 30 to 5°C. Similar strong temperature dependencies (20 to 40-fold increase from to 20 to 5°C) of chemical iron oxidation were observed by others (Roy et al., 2008).

The relative abundance of *Gallionellaceae*, assessed by qPCR and amplicon sequencing, was positively correlated with Fe(II) concentrations both within and across DWTPs. The highest relative abundance of *Gallionella* related OTUs was found at the pre-filter at Aike and Esbjerg DWTP. Although few clones and no DGGE isolates were retrieved of the *Acidovorax* genus, amplicon libraries revealed a significant abundance in RSFs, especially in after filters of Esbjerg and Islevbro DWTP. Cultured Fe(II) oxidizing *Acidovorax* strains are obligate mixotrophs that only utilize Fe(II) in the presence of an organic acid (Muehe et al., 2009), which may explain the lack of *Acidovorax* in our enrichments. In contrast with ESEM micrographs (Figure 1), *Leptothrix* types, known tubular sheath producers (Fleming et al., 2011) were not detected at the Islevbro DWTP. Occurrence of *Leptothrix* has previously been linked to DOC content in the environment, as most *Leptothrix* members may require the DOC to grow heterotrophically or mixotrophically (Fleming et al., 2014, 2018) The closest cultured relative of RSF clones affiliated to *Ferriphaselus* genus was *Ferriphaselus amnicola*, an aerobic, neutrophilic, and stalk-forming Fe(II)-oxidizing bacterium recently isolated from an iron-rich groundwater seep (Kato et al., 2014). FISH results confirmed iron-dependent growth of the detected *Ferriphaselus* phylotypes.

*Undibacterium* and *Curvibacter* IOBs contributed to 4% of the total sequence abundance at Aike, 0.04% at Esbjerg and 0.22% at Islevbro DWTP. *Undibacterium* sequences were more abundant in pre- than after filters, whereas *Curvibacter* sequences showed no clear abundance pattern in RSFs. Abundance of *Undibacterium* IOB ranged from 0.08 to 6% across DWTP, suggesting a wide ecological niche. *Curvibacter* lineage IOB were barely detected in DWTPs with abundance values below 0.5%. Overall, our results indicate *Gallionella* spp. as main contributors to biological iron oxidation at DWTP receiving high Fe(II) concentrations. IOB belonging to *Undibacterium* and *Ferriphaselus* are also important IOB in RSFs, especially in pre-filters receiving influents with high iron concentrations.

## Author Contributions

AG and BS conceived the study. AG performed the research, generate and analyzed the data, and drafted the manuscript. YÇ performed the FISH supported by AG. SM performed the qPCR. AG and BS lead the interpretation of the results. AG, BS, YÇ, SM, and HJ read, edited, and approved the manuscript.

## Conflict of Interest Statement

The authors declare that the research was conducted in the absence of any commercial or financial relationships that could be construed as a potential conflict of interest.

## Footnotes

1 http://www.lgcstandardsatcc.org

2 http://qiime.org/index.html
