# Supplementary material for: Diversity of Iron Oxidizers in Groundwater-Fed Rapid Sand Filters: Evidence of Fe(II)-Dependent Growth by Curvibacter and Undibacterium spp
Source: Front Microbiol. 2018 Dec 3;9:2808. doi: 10.3389/fmicb.2018.02808 (PMC6287000; doi:10.3389/fmicb.2018.02808)
Supplement: Supplementary file 1 [file Data_Sheet_1.pdf]

Supporting information:

Diversity of iron oxidizers in groundwater-fed rapid sand filters: Evidence of Fe(II)-dependent growth by Curvibacter and Undibacterium spp.

Authors: Arda Gülay[1,4*], Yağmur Çekiç[2], Sanin Musovic[3], Hans-Jørgen Albrechtsen[1], Barth F. Smets[1*]

[1] Department of Environmental Engineering, Technical University of Denmark, Denmark.

[2] Department of Environmental Engineering, Istanbul Technical University, Turkey.

[3] Danish Technological Institute, 8000, Århus, Denmark

[4] Department of Organismic and Evolutionary Biology, Harvard University, USA

*Corresponding authors; email: argl@env.dtu.dk; bfsm@env.dtu.dk

Table S1 Influent and effluent water chracteristics at investigated waterworks
Fig.S1 Geographical locations of investigated waterworks
Fig. S2 Process flow of Islevbro DWTP
Fig. S3 Process flow at Aike DWTP.
Fig. S4 Process flow at Esbjerg DWTP
Fig.S5 16S rRNA DGGE profiles of enrichments in highest positive dilutions in the Fe^{2+}/O_2 gradient tubes.
Fig.S6. Phylogenetic tree of bacterial 16S rRNA gene sequences retrieved from enrichments in highest positive dilutions in the $Fe^{2+/}O_2$ gradient tubes series (Fig. S5).
Fig.S7. 16S rRNA DGGE profiles of IOB 10 advanced enrichments
Fig.S8 Liquid incubations inoculated with enrichments from highest positive dilutions
Fig.S9 Hybridization efficiency curves of designed probes retrieved by MathFISH
Fig.S10 Quantification of the hybridized cell number and mean fluorencence intensity
Fig.S11 Neighbor-joining phylogenetic tree of large subunit of ribulose-1,5-bisphosphate carboxylase/oxygenase (RuBisCO) genes (PF02788) retrieved from pfam database.
Table S2 Culture-based estimates of IOB and qPCR-based estimates of *Gallionella* spp. per gr of filter material at investigated waterworks
Table S3 Taxonomic classification of 16S rRNA sequences from the DGGE bands from selected enrichments (marked as "+" in Figure S5) in highest positive dilutions in the $Fe^{2+/}O_2$ gradient tubes. Selected enrichments contain single bands in their DGGE profiles.
Table S4. Probes used for the detection of target organisms by *in situ* fluorescent hybridization

Table S1 Influent and effluent water chracteristics at investigated waterworks

		**Esbjerg**[a]		**Islevbro**[b]		**Aike**[c]	
		Influent	Effluent	Influent	Effluent	Influent	Effluent
H_2S	mg/L	<0.020	<0.005	0.02	<0.005	-	<0.02
CH_4	mg/L	<0.005	<0.005	0.03	<0.005	-	<0.01
O_2	mg/L	0.6	10	1.1	8.6	0.9	10
Fe	mg/L	12	0.019	1.07	0.03	1.8	0.017
Mn	mg/L	0.3	<0.005	0.06	0.002	0.63	<0.005
pH	mg/L	6.9	8	7.4	7.2	7.5	7.8
°C	celcius	8.5	8.5	8.5	8.4	8.2	8.3

[a-b] Influent and effluent water qualities were measured on site

[c] Water quality parameters were determined from previous reports

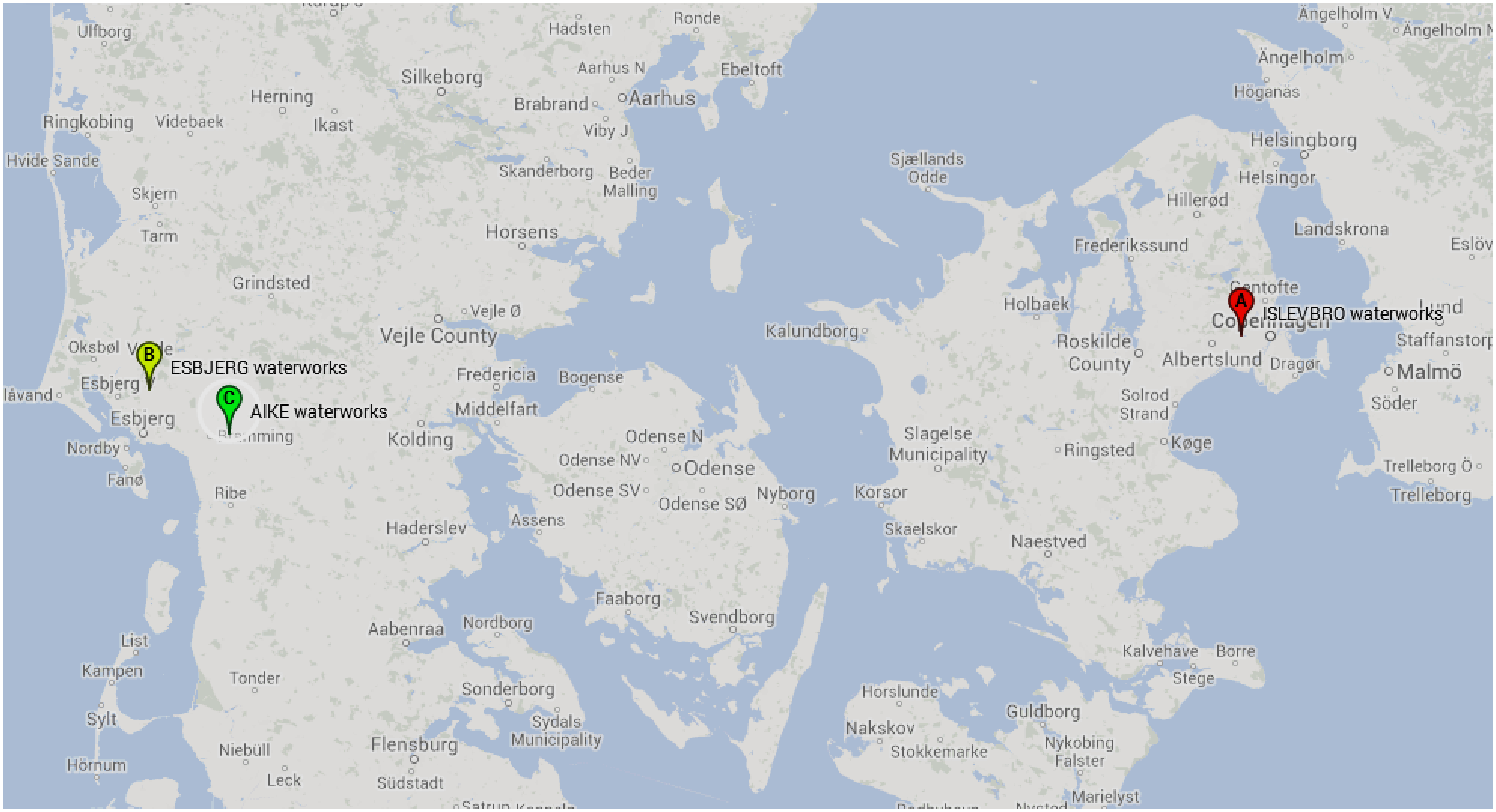

Fig.S1 Geographical locations of investigated waterworks

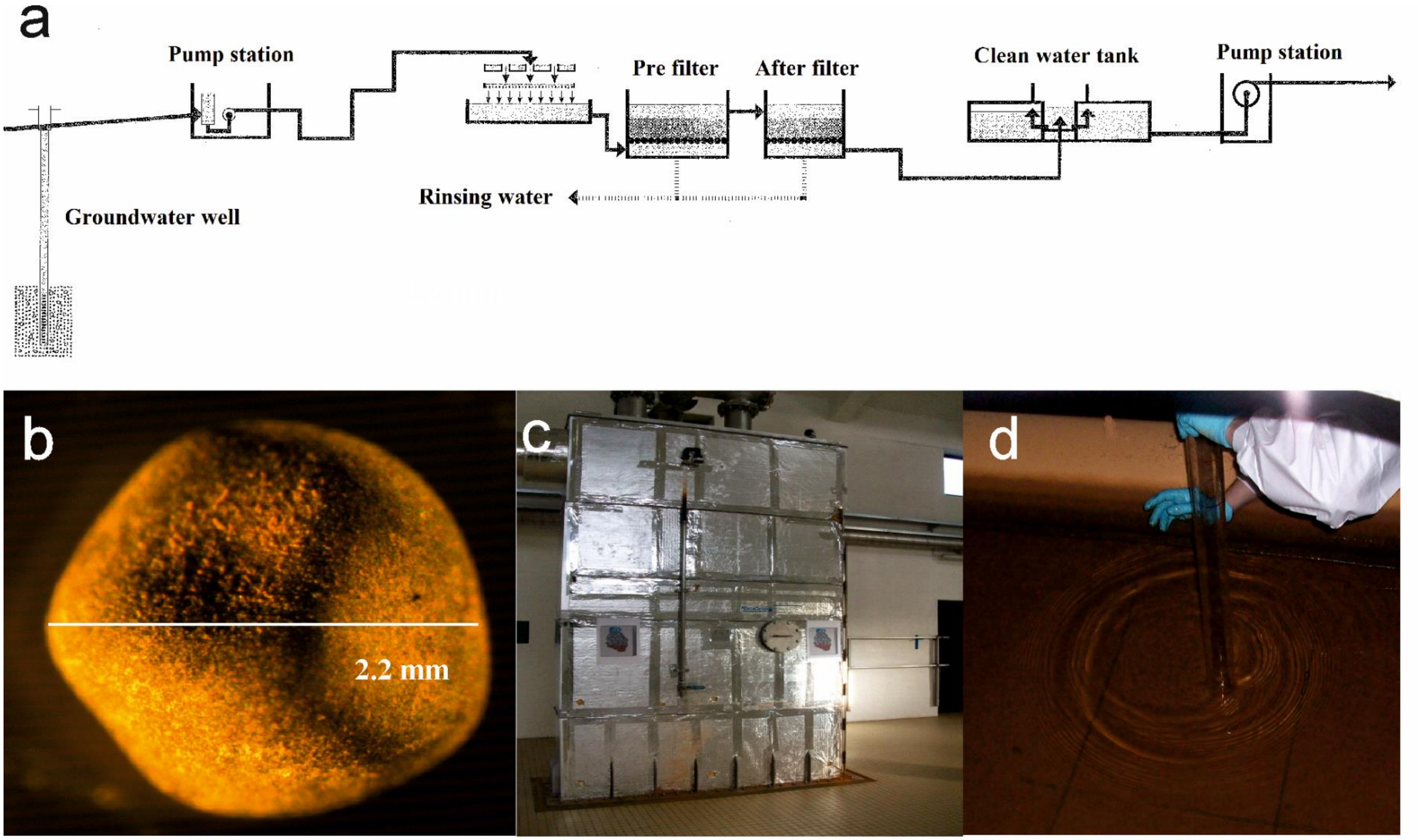

Fig. S2 Process flow of Islevbro DWTP (a), a typical after filter sand grain from the top filter layer (0-10 cm) (b), aeration chamber (c), a represantative core sampling effort from an after filter at Islevbro DWTP.

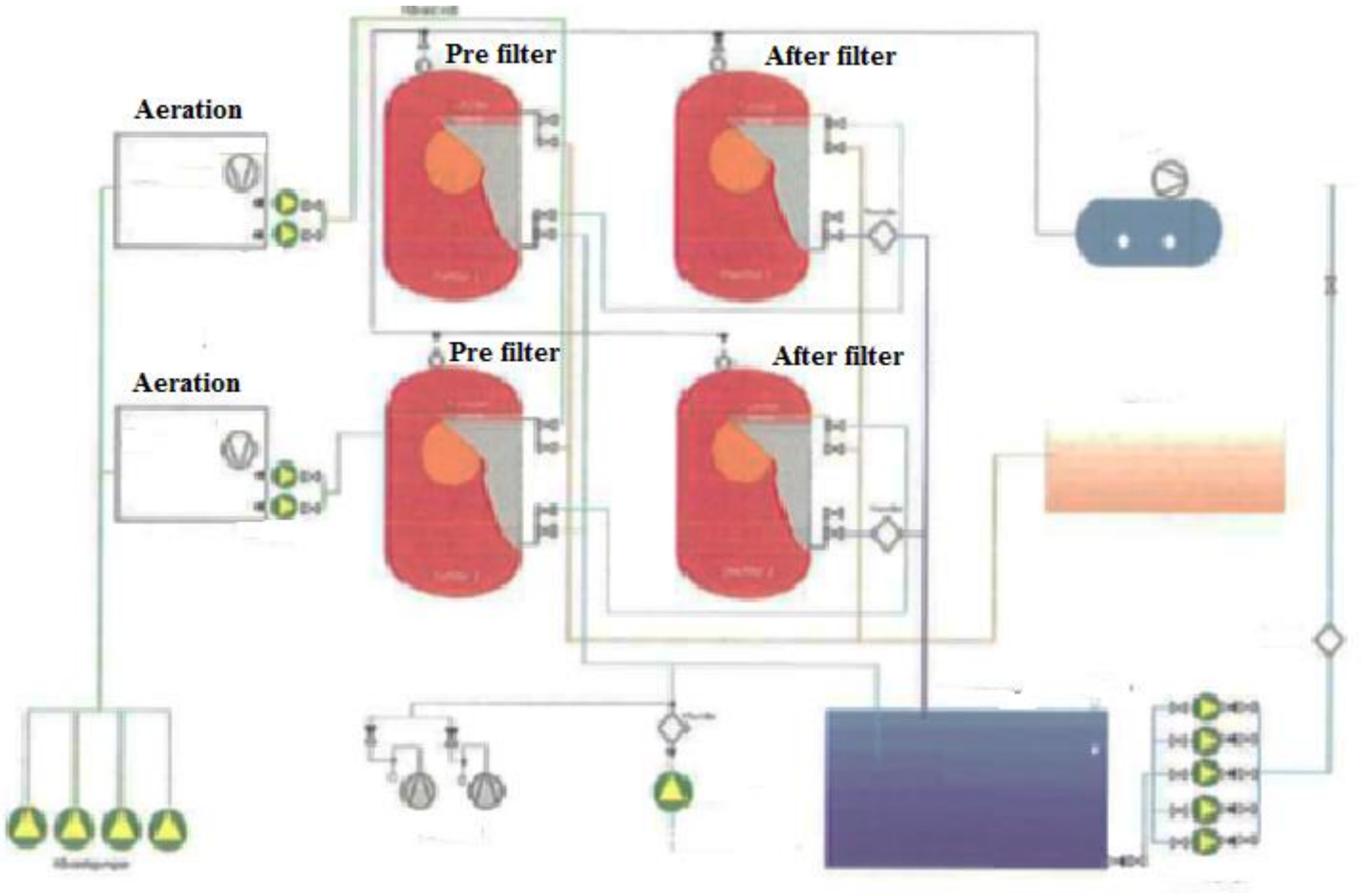

Fig. S3 Process flow at Aike DWTP. All filters are pressurized sand filters.

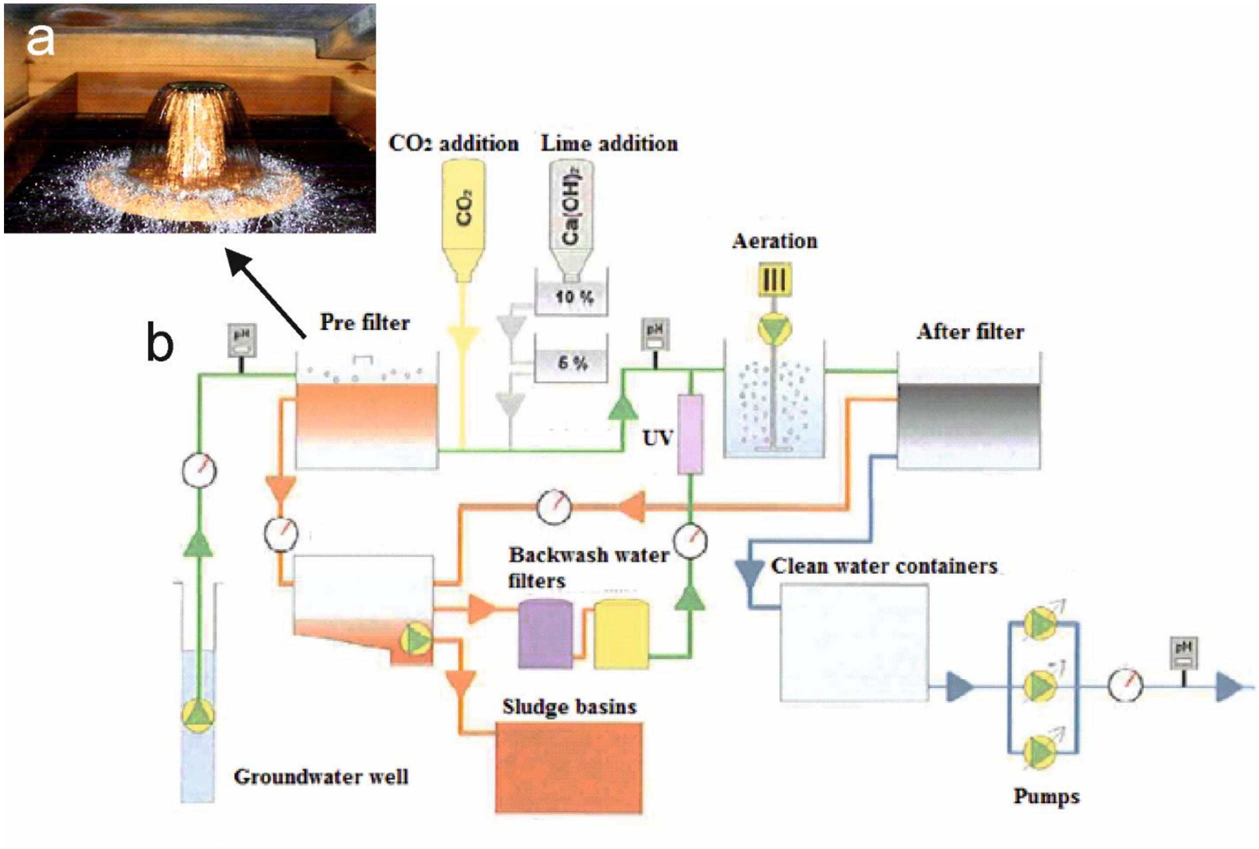

Fig. S4 Process flow at Esbjerg DWTP (b). Light aeration of anoxic groundwater using a plate aerator in pre filters is done to to enhance biological iron oxidation works (a).

Table S2 Culture-based estimates of IOB and qPCR-based estimates of *Gallionella* spp. per gr of filter material at investigated waterworks

Water works	Units	MPN based total IOB		qPCR based Gallionela spp.	
		Mean (cells gr wet sand^{-1})	SD log_{10}	Mean (gene gr wet sand^{-1})	SD log_{10}
Islevbro	Pre filter	1.80×10^4	0.33	3.07×10^7	6.23
	After filter	5.30×10^4	0.24	3.33×10^7	6.50
	After filter	1.10×10^4	0.33	2.83×10^6	5.11
	After filter	7.10×10^5	0.34	2.71×10^7	5.53
	After filter	1.80×10^4	0.32	2.26×10^7	6.34
Esbjerg	Pre filter	5.80×10^6	0.38	4.49×10^8	7.14
	Pre filter	6.00×10^7	0.39	9.92×10^8	6.96
	Pre filter	1.60×10^7	0.41	4.24×10^8	6.86
	After filter	2.30×10^6	0.32	6.76×10^8	7.76
	After filter	1.10×10^6	0.33	2.45×10^7	6.14
	After filter	1.10×10^6	0.33	5.43×10^7	6.51
Aike	Pre filter	2.30×10^6	0.32	4.05×10^7	6.83
	Pre filter	3.80×10^6	0.27	3.07×10^7	6.23
	Pre filter	3.80×10^6	0.27	4.78×10^7	6.69
	After filter	5.30×10^6	0.24	3.79×10^7	5.07

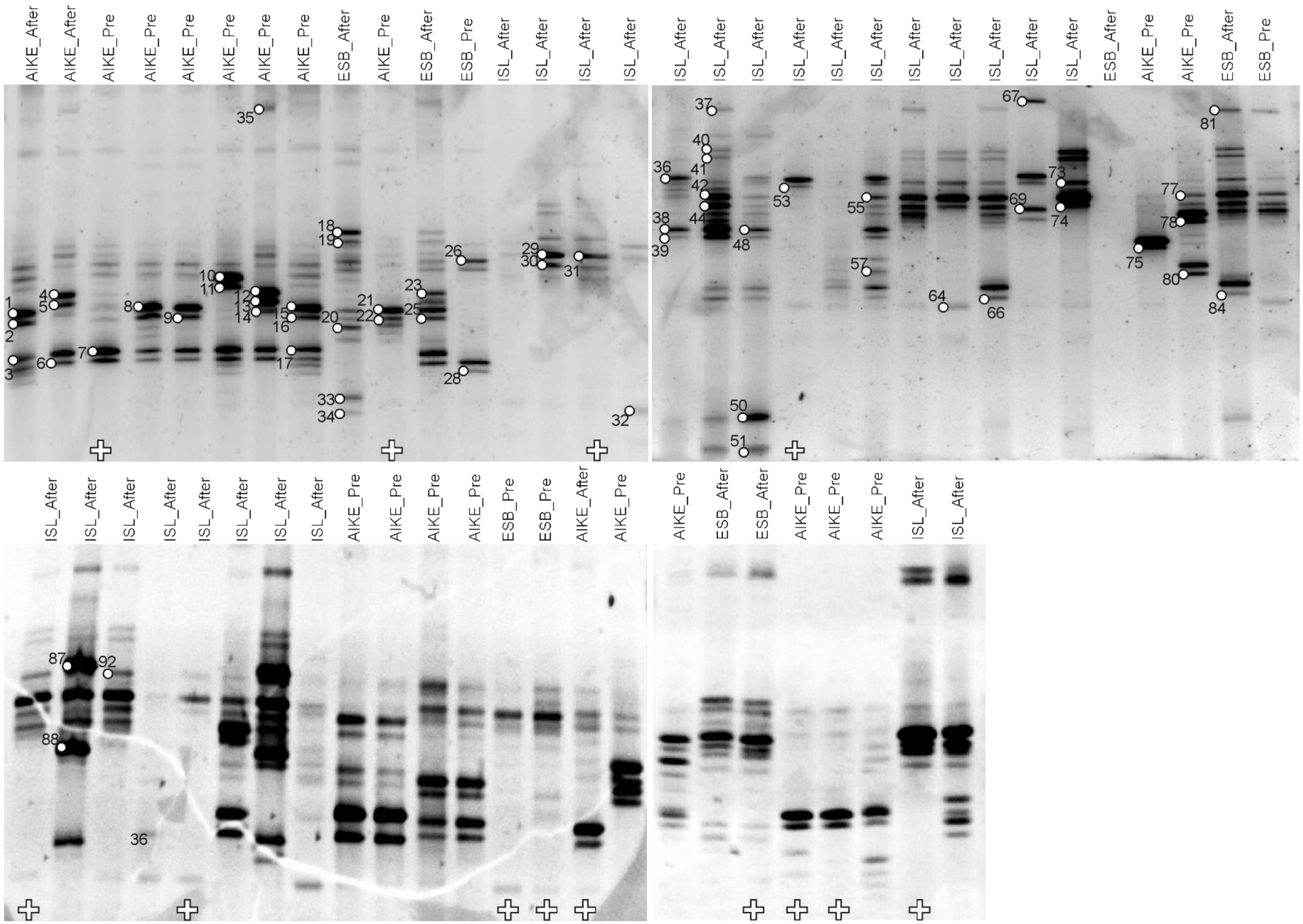

Fig.S5 16S rRNA DGGE profiles of enrichments in highest positive dilutions in the Fe^{2+}/O_2 gradient tubes. Dominant bands were isolated from DGGE gels, re-amplified with 341F and 518R primers, and sequenced (Phylogenetic tree in Fig.S6). Enrichments with DGGE profiles displaying a clear singular dominant band (marked as "+") were selected and PCR amplified using 27F and 1492R primers and sequenced (Taxonomic identities in Table.S3).

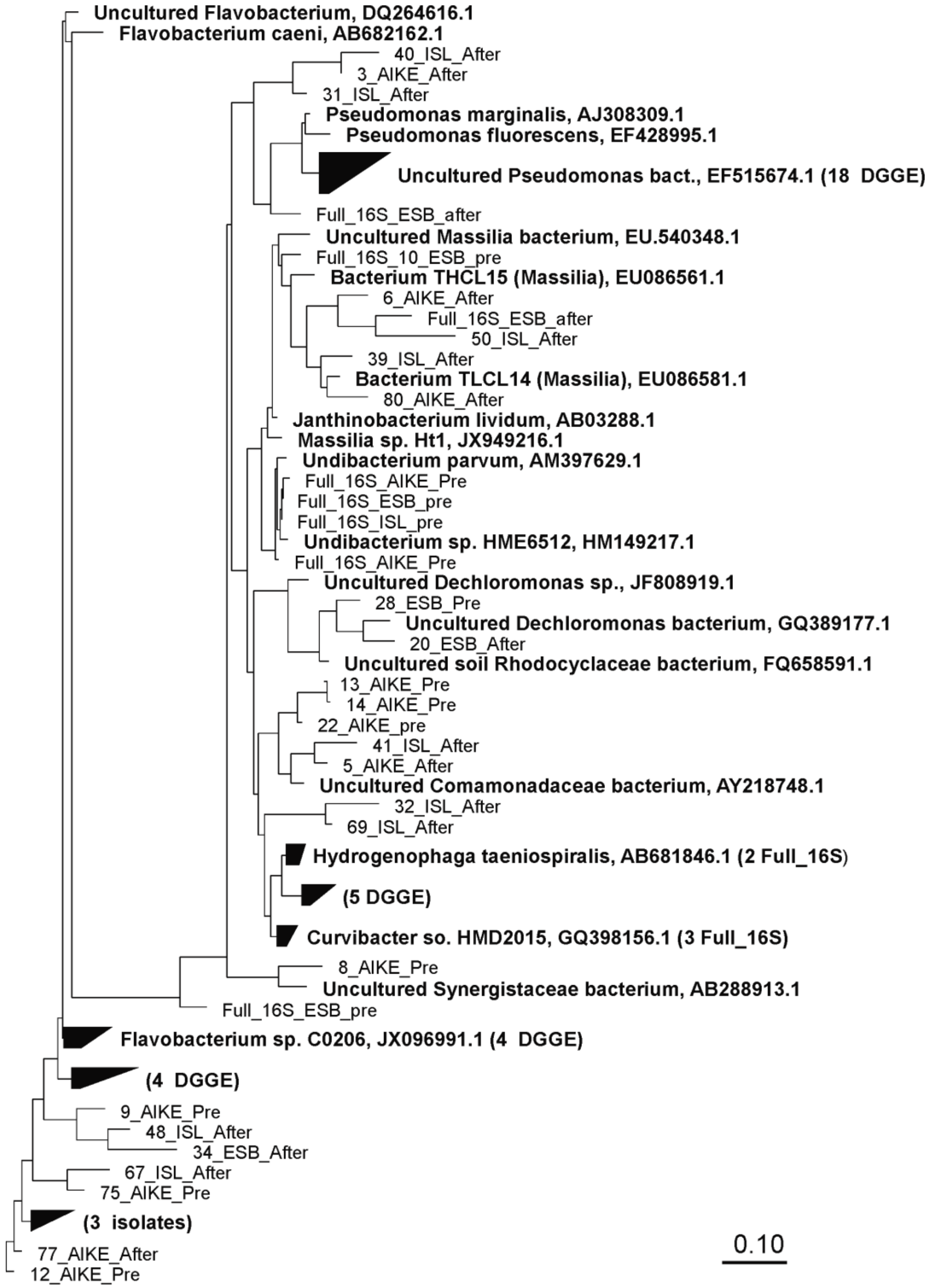

Fig.S6. Phylogenetic tree of bacterial 16S rRNA gene sequences retrieved from enrichments in highest positive dilutions in the Fe^{2+}/O_2 gradient tubes series (Fig. S5). The tree was constructed using neighbor-joining method in ARB software from 170 bp sequences derived by DGGE technique. Numbers shown in parenthesis correspond to the amount of phylotypes in associated ARB clade. The bar indicates 10% sequence difference.

Table S3 Taxonomic classification of 16S rRNA sequences from the DGGE bands from selected enrichments (marked as "+" in Figure S5) in highest positive dilutions in the Fe^{2+}/O_2 gradient tubes. Selected enrichments contain single bands in their DGGE profiles.

DWTP	Filter	Closest cultured relative	Similarity	ID	Family	Genus
Aike	Pre	Undibacterium parvum	99%	AM397629.1	Oxalobacteraceae	Undibacterium
Aike	Pre	Herminiimonas sp. IW-225	98%	KF556700.1	Oxalobacteraceae	Herminiimonas
Aike	After	Curvibacter putative symbiont of Hydra magnipapillata	98%	FN543107.1	Comamonadaceae	Curvibacter
Aike	After	Janthinobacterium sp. MDT1-19	99%	JX949578.1	Comamonadaceae	Janthinobacterium
ESB	Pre	Pseudomonas fluorescens	100%	KF923836.1	Pseudomonadaceae	Pseudomonas
ESB	Pre	Undibacterium sp. HME6512	99%	HM149217.1	Oxalobacteraceae	Undibacterium
ESB	Pre	Rhodoferax sp. enrichment culture clone Van18	99%	HQ222266.1	Comamonadaceae	Rhodoferax
ESB	Pre	Uncultured Janthinobacterium sp. clone ZL30	100%	KC433642.1	Comamonadaceae	Janthinobacterium
ESB	After	Pseudomonas sp. Ln4B.8	100%	KC433642.1	Pseudomonadaceae	Pseudomonas
ESB	After	Janthinobacterium sp. Man12	90%	AY788973.1	Comamonadaceae	Janthinobacterium
ESB	After	Bacterium TLCL14	92%	EU086581.1	Comamonadaceae	Massilia
ISL	Pre	Herminiimonas sp. IW-225	99%	KF556700.1	Oxalobacteraceae	Herminiimonas
ISL	After	Hydrogenophaga sp. Rs71	99%	AM110076.2	Comamonadaceae	Hydrogenophaga
ISL	After	Bacterium BH3O1	99%	AY928221.1	Comamonadaceae	Albidiferax
ISL	After	Rhodoferax sp. Asd M2A	99%	FM955857.1	Comamonadaceae	Rhodoferax

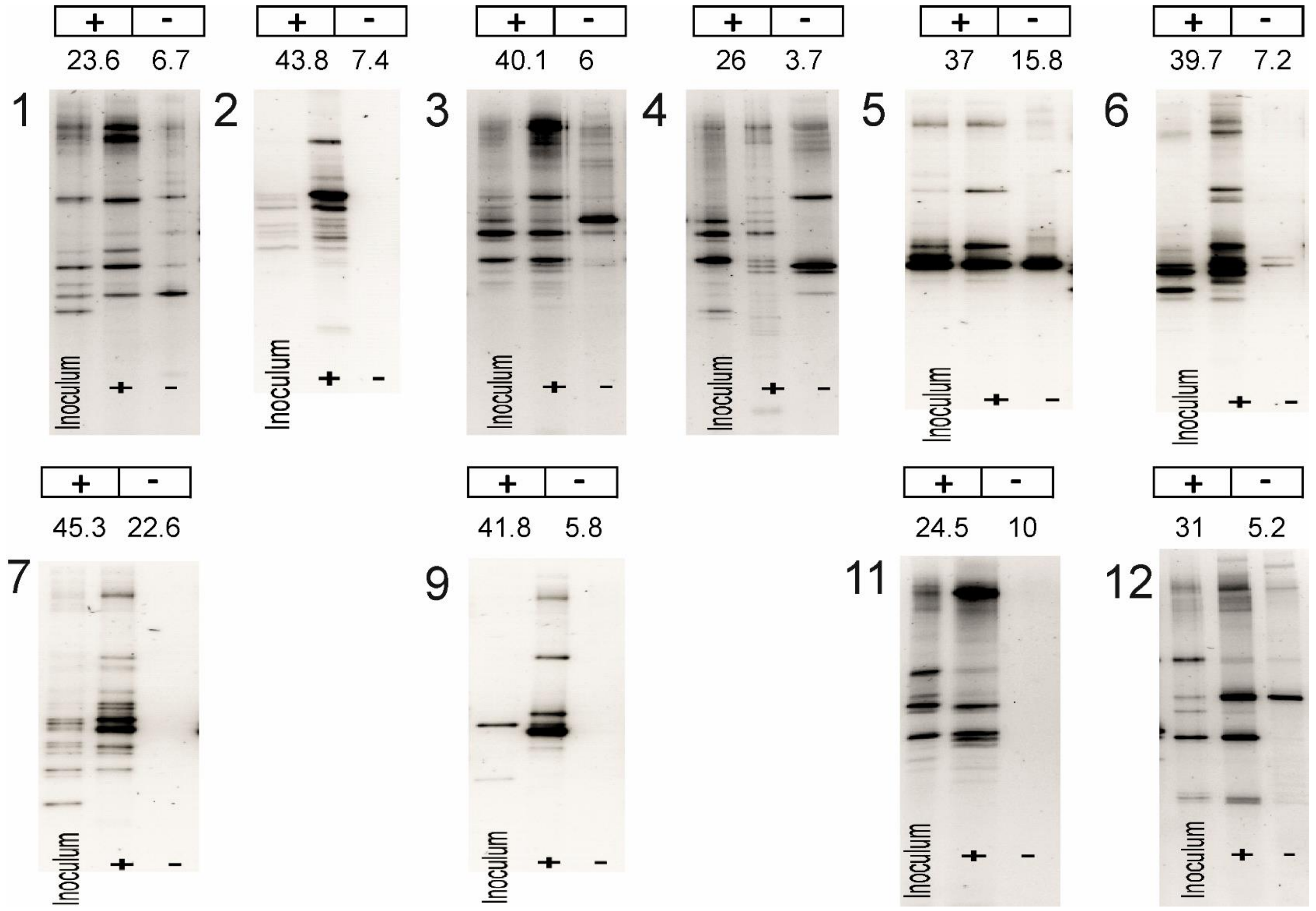

Fig.S7. 16S rRNA DGGE profiles of IOB 10 advanced enrichments taken from Fe^{2+}/O_2 gradient tubes (Fig.2, step 4) and from subsequent liquid batch incubations (Fig.2, step 5) in presence (+) or absence (-) of Fe^{2+}. Total extractable DNA (ng/µl) after 15 days incubations. Empty DGGE profiles indicate low amplicon yields at standard PCR conditions

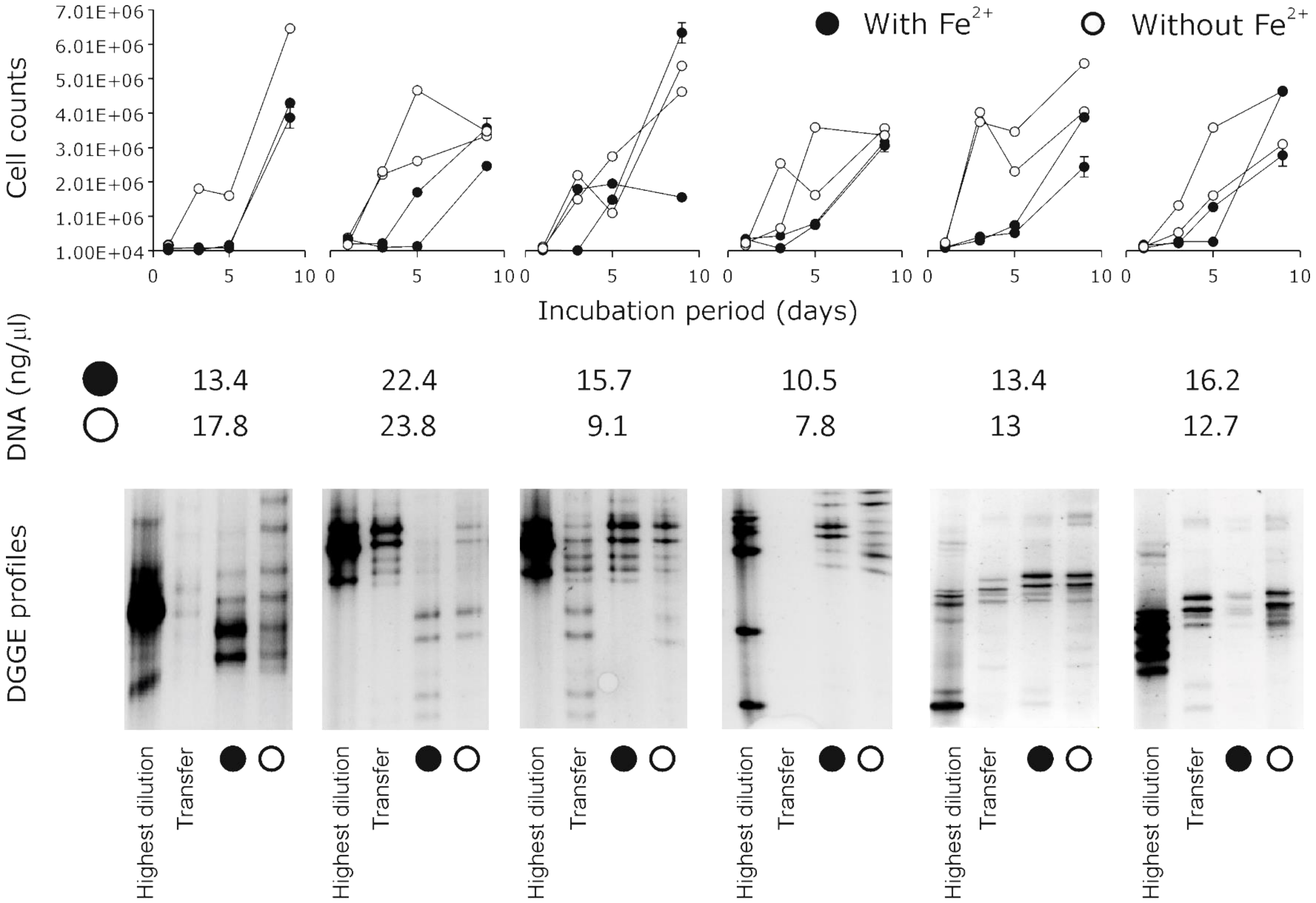

Fig.S8 Liquid incubations inoculated with enrichments from highest positive dilutions (56 samples in Fig.S5) in the Fe^{2+}/O_2 gradient tubes to verify Fe^{2+} stimulated growth. Graphs show the cell number increase during 12 days incubations in the presence (black circles) and absence (white circles) of Fe^{2+}**.** 16S rRNA DGGE profiles taken from the Fe^{2+}/O_2 gradient tubes ("Highest dilution"), from transferred highest positive dilution cultures ("Transfer") and from subsequent liquid cultivations in presence (●) or absence (○) of Fe^{2+}.

Undibacterium probe

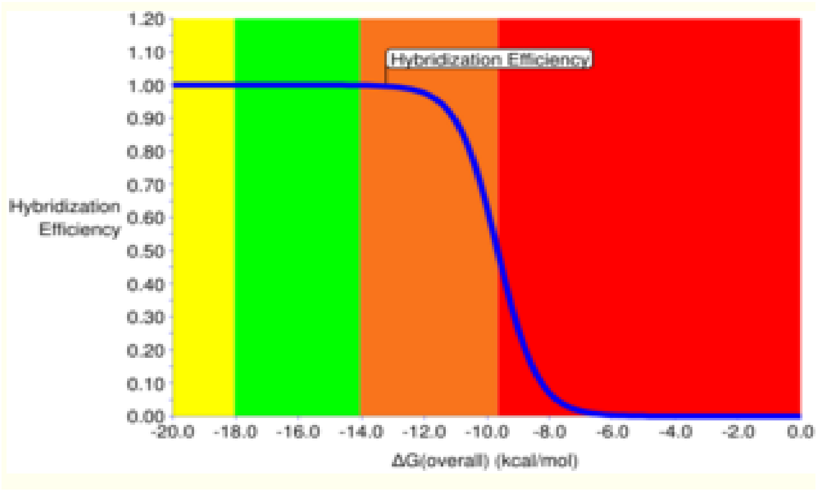

Curvibacter probe

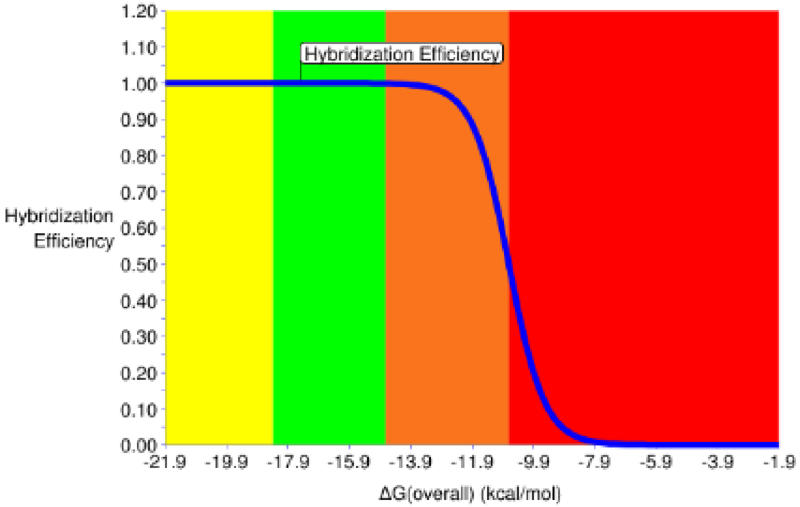

Ferriphaselus probe

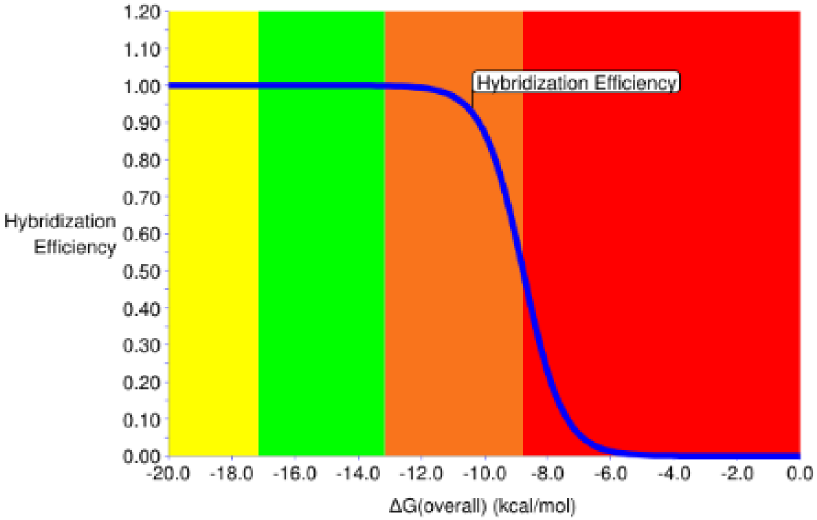

Rhodoferax probe

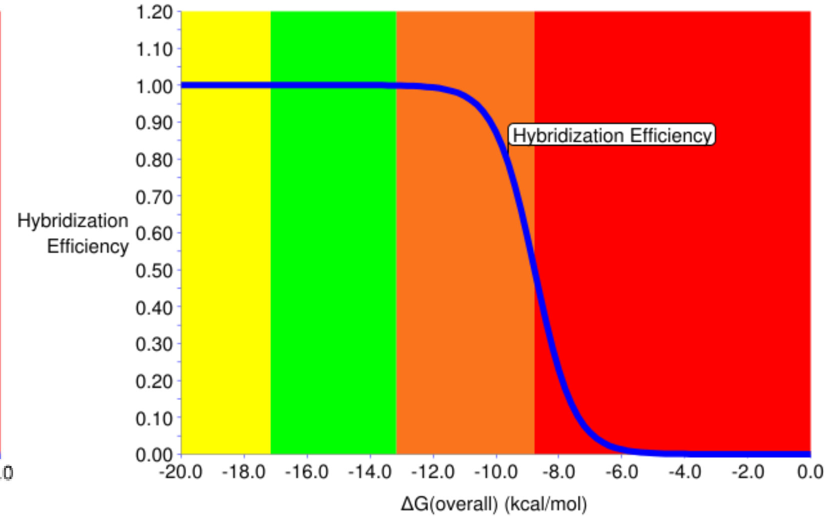

Fig.S9 Hybridization efficiency curves of designed probes retrieved by MathFISH, an online web tool that uses thermodynamics-based mathematical models for in silico evaluation of oligonucleotide probes for FISH

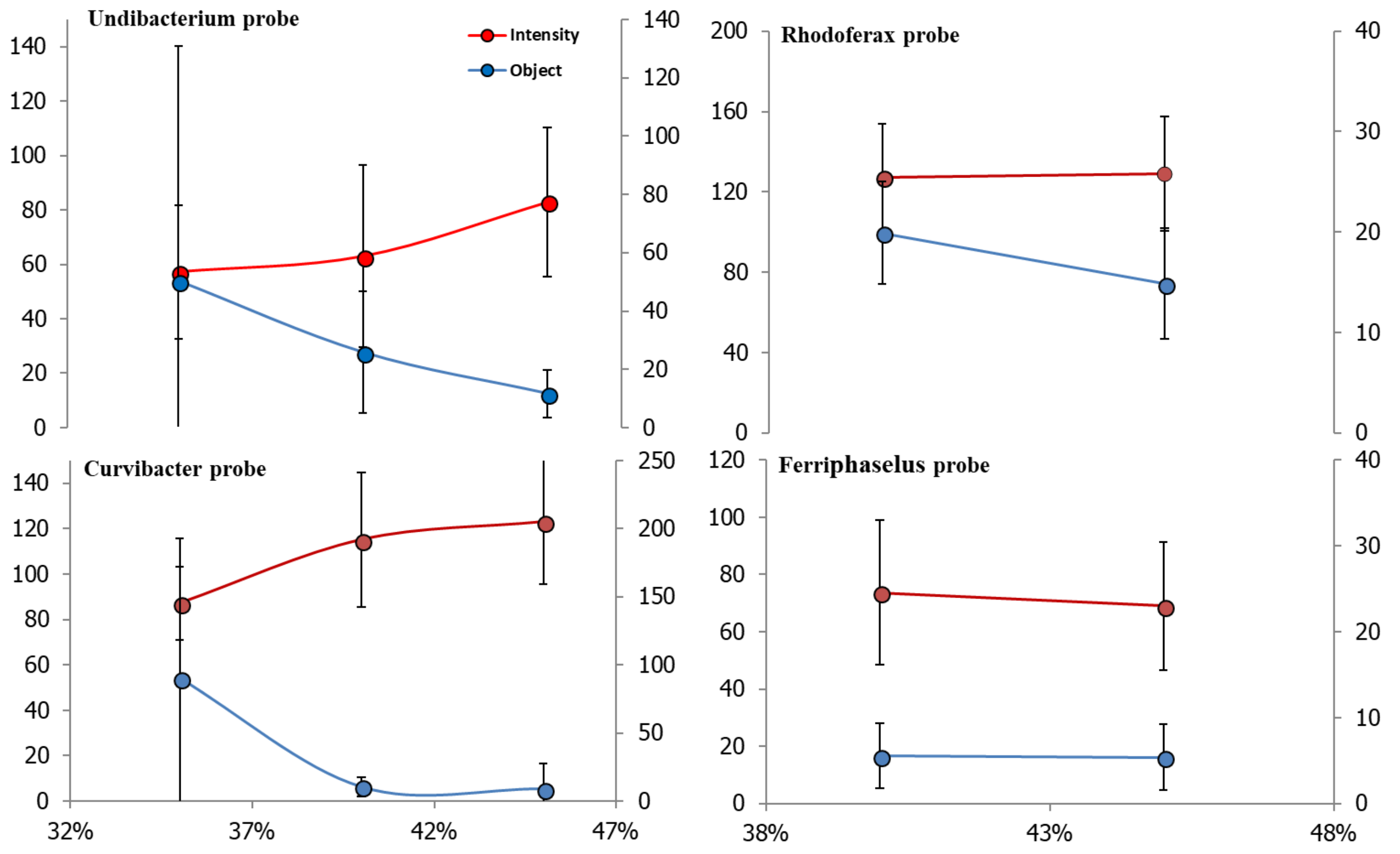

Fig.S10 Quantification of the hybridized cell number and mean fluorencence intensity under increasingly stringent hybridization and washing conditions to determine optimum formamide concentrations. For each data point the mean fluorescence intensity of at least 50 cells was determined. Error bars indicate the standard deviation.

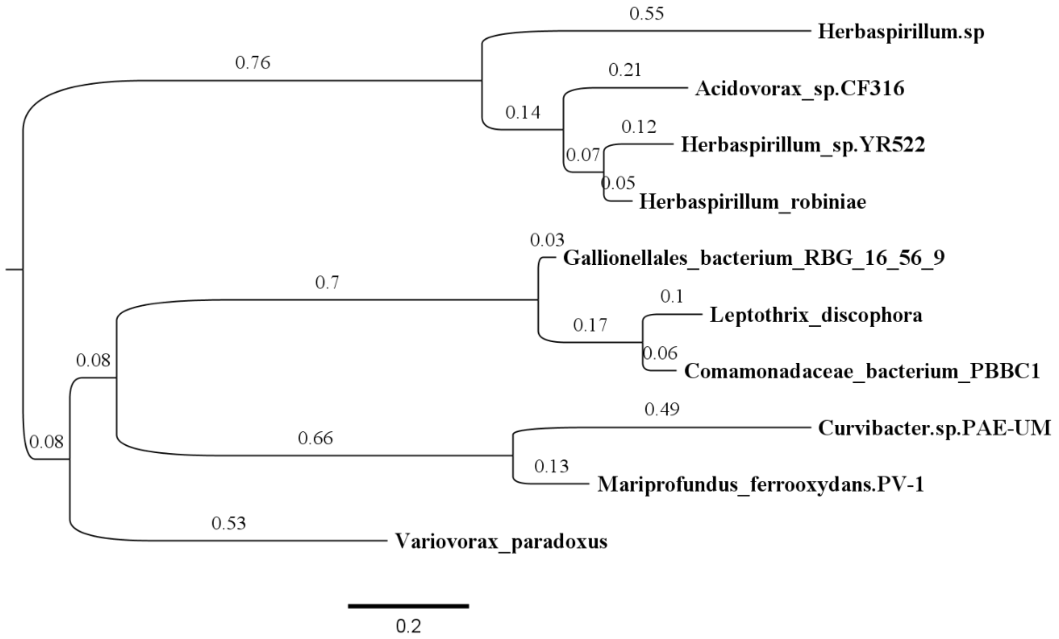

Fig.S11 Neighbor-joining phylogenetic tree of large subunit of ribulose-1,5-bisphosphate carboxylase/oxygenase (RuBisCO) genes (PF02788) retrieved from pfam database. Sequences were aligned using Muscle and the trees were constructed by ClustalW. The scale bar represents 0.2 substitutions per nucleotide position.

Table S4. Probes used for the detection of target organisms by *in situ* fluorescent hybridization

Probe name	Target	Probe sequence (5'-3')	FA (%)	Position*	Reference
EUB338	Most Bacteria	GCT GCC TCC CGT AGG AGT	35	338	(Amann et al., 1990)
EUB338 II	*Planctomycetales*	GCA GCC ACC CGT AGG TGT	35	338	(Daims et al., 1999)
EUB338 III	*Verrucomicrobiales*	GCT GCC ACC CGT AGG TGT	35	338	(Daims et al., 1999)
UNDI_453	*Undibacterium*	5'- GTG TAT TAG ACC TCA CCG -3' – Cy3	45	453	This study
CURVI_454	*Curvibacter*	5'- CTC ACC GTT TCG TTC CGT -3' – Cy5	40	454	This study
RHODO_445	*Rhodoferax*	5'- GAA GAG ACC GTT TCG TTC -3' – Cy5	45	445	This study
FERRI_453	*Ferriphaselus*	5'- CCA CAC TCC GTA TTA GGA -3-Cy3	40	453	This study

* Position on the *E. coli* 16S rRNA sequence
